# Supplementary material for: Effect of Different Formulations and Storage on the Physicochemical, Microbiological, and Organoleptic Characteristics of Dovyalis caffra Fruit Yogurt
Source: Foods. 2024 Dec 18;13(24):4102. doi: 10.3390/foods13244102 (PMC11675328; doi:10.3390/foods13244102)
Supplement: Supplementary file 1 [file foods-13-04102-s001.zip › foods-3307437-supplementary.pdf]

Table S1. Effect of storage on the color parameters of *Dovyalis caffra* fruit yoghurt

Parameter	Product	Storage period			
		Day 1	Day 7	Day 14	Day 21
L*	D-0	81.42±3.69A,a	80.66±3.59a	80.48±3.70a	79.88±2.02a
	D-5	79.04±3.73A,a	78.22±3.92a	78.25±3.71a	78.42±1.66a
	D-10	78.74±3.23A,a	78.30±2.44a	76.71±4.63a	77.27±3.87a
	D-15	78.59±0.82A,a	78.72±1.52a	77.81±1.10a	77.57±2.94a
a*	D-0	-0.21±0.14A,a	-0.20±0.09a	-0.21±0.06a	-0.21±0.07a
	D-5	-0.23±0.12A,a	-0.24±0.14a	-0.21±0.17a	-0.24±0.06a
	D-10	-0.27±0.11A,a	-0.32±0.12a	-0.32±0.04a	-0.30±0.13a
	D-15	-0.32±0.18A,a	-0.37±0.22a	-0.34±0.19a	-0.36±0.16a
b*	D-0	9.26±0.79A,a	9.36±0.09a	9.06±0.08a	8.85±1.37a
	D-5	18.43±0.08B,a	18.08±0.20a	17.95±0.08a	18.11±0.74a
	D-10	22.86±0.31C,a	22.45±1.97a	23.02±0.08a	23.67±0.55a
	D-15	25.28±1.02C,a	25.27±0.55a	25.40±0.08a	24.62±0.31a
C*	D-0	9.26±0.80A,a	9.37±0.09a	9.06±0.08a	8.85±1.37a
	D-5	18.44±0.08B,a	18.08±0.20a	17.95±0.08a	18.11±0.74a
	D-10	22.86±0.31C,a	22.45±1.97a	23.03±0.08a	23.68±0.54a
	D-15	25.28±1.03D,a	25.27±0.55a	25.41±0.08a	24.62±0.32a
h*	D-0	91.34±0.99A,a	91.26±0.57a	91.33±0.37a	91.38±0.63a
	D-5	90.72±0.39A,a	90.75±0.37a	90.67±0.54a	90.78±0.19a
	D-10	90.68±0.28A,a	90.86±0.29a	90.80±0.11a	90.73±0.29a
	D-15	90.74±0.45A,a	90.85±0.53a	90.78±0.43a	90.72±0.19a

Data presented as means ± standard deviation, Means in columns within a specific parameter with the same upper case superscript are not significantly different (P>0.05), Means in rows with the same lower case superscript are not significantly different (P>0.05)

Table S2. Effect of storage on the sensory parameters of *Dovyalis caffra* fruit yoghurt

Parameter	Product	Storage period			
		Day 1	Day 7	Day 14	Day 21
Colour	D-0	7.23±0.23^{A,a}	7.18±0.20^{a}	7.20±0.33^{a}	7.21±0.15^{a}
	D-5	7.23±0.28^{A,a}	7.18±0.15^{a}	7.22±0.30^{a}	7.14±0.41^{a}
	D-10	7.18±0.15^{A,a}	7.18±0.10^{a}	7.12±0.12^{a}	7.09±0.07^{a}
	D-15	6.84±0.63^{A,a}	6.70±0.48^{a}	6.75±0.51^{a}	6.70±0.48^{a}
Taste	D-0	7.94±0.02^{D,c}	7.77±0.03^{b}	7.73±0.03^{b}	6.69±0.02^{a}
	D-5	7.38±0.12^{C,b}	7.11±0.10^{ab}	6.70±0.13^{a}	6.61±0.15^{a}
	D-10	6.02±0.03^{B,c}	6.04±0.05^{c}	5.83±0.05^{b}	5.29±0.05^{a}
	D-15	5.09±0.13^{A,c}	4.84±0.03^{bc}	4.77±0.03^{b}	4.09±0.03^{a}
Texture	D-0	7.61±0.10^{D,b}	7.50±0.10^{b}	7.29±0.10^{b}	6.43±0.10^{a}
	D-5	7.12±0.12^{C,b}	6.97±0.10^{b}	6.84±0.13^{ab}	6.54±0.10^{a}
	D-10	6.34±0.07^{B,b}	6.22±0.10^{b}	6.07±0.15^{ab}	5.68±0.10^{a}
	D-15	5.62±0.12^{A,b}	5.31±0.08^{b}	5.25±0.10^{b}	4.82±0.05^{a}
Overall acceptability	D-0	8.21±0.15^{C,a}	7.95±0.18^{a}	7.73±0.23^{a}	7.66±0.18^{a}
	D-5	7.47±0.20^{B,a}	7.40±0.21^{a}	7.27±0.23^{a}	7.18±0.20^{a}
	D-10	7.20±0.23^{B,a}	7.18±0.20^{a}	7.11±0.26^{a}	7.00±0.25^{a}
	D-15	5.23±0.18^{A,b}	5.11±0.20^{ab}	4.77±0.27^{ab}	4.25±0.25^{a}

Data presented as means ± standard deviation, Means in columns within a specific parameter with the same upper case superscript are not significantly different (P>0.05), Means in rows with the same lower case superscript are not significantly different (P>0.05)

Table S3. Microbial load in *Dovyalis caffra* fruit yoghurt

Parameter	Product	Storage period			
		Day 1	Day 7	Day 14	Day 21
Total yeast and mould count (cfu/g)	D-0	ND	ND	ND	ND
	D-5	ND	ND	ND	ND
	D-10	ND	ND	ND	ND
	D-15	ND	ND	ND	ND
Total coliforms count (cfu/g)	D-0	ND	ND	ND	ND
	D-5	ND	ND	ND	ND
	D-10	ND	ND	ND	ND
	D-15	ND	ND	ND	ND

ND = Not Detected
